# Rain triggers seasonal stratification in a temperate shelf sea

**DOI:** 10.1038/s41467-023-38599-y

**Published:** 2023-06-02

**Authors:** J. E. Jardine, M. Palmer, C. Mahaffey, J. Holt, S. L. Wakelin, A. Düsterhus, J. Sharples, J. Wihsgott

**Affiliations:** 1 National Oceanography Centre, L3 5DA Liverpool, UK; 2 Department of Earth, Ocean and Ecological Sciences, School of Environmental Sciences, L69 3GP Liverpool, UK; 3 Plymouth Marine Laboratory, PL1 3DH Plymouth, UK; 4 Irish Climate Analysis and Research UnitS (ICARUS), Department of Geography, Maynooth University, Co. Kildare, Ireland

**Keywords:** Physical oceanography, Climate and Earth system modelling

## Abstract

The North Atlantic Storm Track acts as a conveyor belt for extratropical cyclones that frequently deliver high winds and rainfall to northwest European shelf seas. Storms are primarily considered detrimental to shelf sea stratification due to wind-driven mixing countering thermal buoyancy, but their impact on shelf scale stratification cycles remains poorly understood. Here, we show that storms trigger stratification through enhanced surface buoyancy from rainfall. A multidecadal model confirms that rainfall contributed to triggering seasonal stratification 88% of the time from 1982 to 2015. Stratification could be further modulated by large-scale climate oscillations, such as the Atlantic Multidecadal Variability (AMV), with stratification onset dates being twice as variable during a positive AMV phase than a negative one. Further insights into how changing storm activity will impact shelf seas are discussed beyond the current view of increasing wind-driven mixing, with significant implications for marine productivity and ecosystem function.

## Abstract (web summary)

Seasonal stratification on the northwest European Shelf is found to be triggered by rainfall from passing storms. Further links are made between the onset of stratification to large-scale pressure changes in the North Atlantic.

## Introduction

Northwest (NW) Europe is positioned directly beneath the North Atlantic Storm Track and experiences extratropical cyclones year-round^1^, with the strongest storms usually occurring during the winter months^2^. The intensity and frequency of these storms have immense economic and societal impacts, with the winters of 2013-14 and 2015-16 highlighted as being particularly destructive storm seasons that caused widespread damage to coastal defences and infrastructure^3–5^. The impact of storm variability on the marine environment is considerably less well studied.

In temperate shelf seas, the seasonal transition from well-mixed waters during winter to summer stratified conditions acts as a precursor to the rapid growth of phytoplankton known as the spring bloom^6–9^. This phytoplankton growth event accounts for up to one third of the total annual primary productivity on the shelf^10^. The timing of the spring bloom is important for the phenology of zooplankton and fish larvae, with implications for the supply of food to higher trophic levels and the spawning success of fish stock^11,12^. Given that, globally, 4.5 billion people rely on fish for an estimated 15% of their protein intake^13^ and shelf seas account for 90% of the global fish catch^14^, it is imperative to understand how climate influences both the timing of stratification and the development of the spring bloom, and how these will change in future climate scenarios.

In temperate shelf seas, a combination of net cooling, tidal stirring and strong winds act to mix the water column during winter months, resulting in a vertically homogenous distribution of high nutrient concentrations and low concentrations of phytoplankton cells. This, combined with seasonally lower irradiance during the winter months, results in limited phytoplankton growth^6^. The canonical view of temperate shelf sea stratification and spring bloom initiation is that increasing solar irradiance in spring leads to net surface heat input, which eventually overcomes the wind and tidal mixing to develop thermal stratification^15–17^. Phytoplankton trapped in the nutrient-rich surface layer then receive sufficient light to grow quickly and form the spring bloom^7,9^.

Rainfall has been shown to promote stratification through surface freshwater addition in subtropical^18,19^ and monsoonal regions^20^. But while rainfall has been postulated to contribute to stratification within temperate shelf seas^7^ and may support episodic phytoplankton growth^21^, it has largely been discounted as a critical controlling mechanism for the onset of seasonal stratification as it is considered a negligible buoyancy input compared to thermal heating^22–24^. A lack of observations at high spatial and temporal resolutions during winter has been a historical barrier to forming increased understanding of winter ocean conditioning.

Autonomous robotic ocean gliders^25^ provide a valuable contribution to ocean observing that has the potential to fill this knowledge gap. Ocean gliders can sample through adverse weather conditions and resolve near surface processes away from the potential contamination of large research vessels or traditional fixed ocean platforms. In this study, we investigated the onset of seasonal stratification in the Celtic Sea in March 2015 using data collected by a glider deployed as part of the UK Shelf Sea Biogeochemistry project (www.uk-ssb.org). As the glider captured the subtle ocean-atmosphere coupling from passing storm events, we then quantified the importance of rainfall as the initial triggering mechanism for seasonal stratification in this region. Finally, we used a multi-decadal 3D model to infer the linkages between stratification onset in the Celtic Sea and large-scale modes of climate variability across the North Atlantic.

## Results

**Fig. 1:**
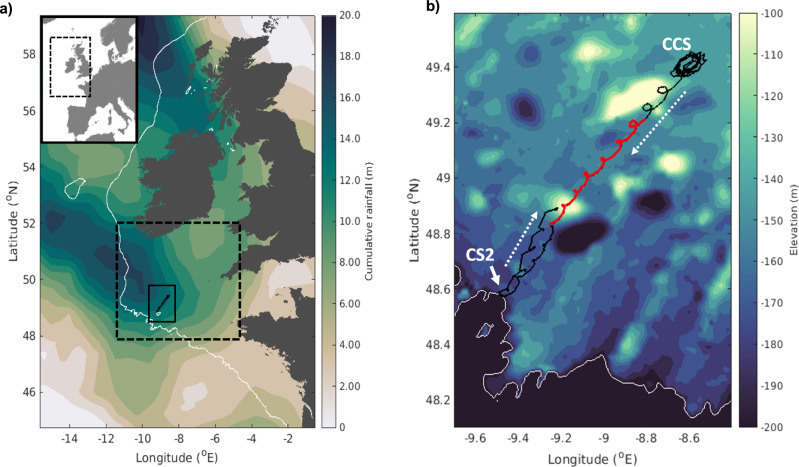
Study site. **a** Location of the glider track (solid black line) in relation to the North West (NW) European Shelf (inset map) and the wider Celtic Sea region (dashed black box), including the accumulated rainfall in mm (colour map) over 24 hours from 12:00UTC on the 25th March 2015 to 12:00UTC on the 26th March 2015 (ERA-Interim^38^); **b** A close up of the glider track (defined by the solid red box in **a**) over bathymetry (colour map), with the solid black line denoting the entire glider track from the 22nd March to the 2nd April 2015. The stratification event from 25th - 29th March 2015 is denoted by the red part of the track. The direction of glider travel (dashed white arrows), the location of the Central Celtic Sea mooring site (CCS) and the shelf break site (CS2), and the 200 m depth contour indicative of the shelf break (white contour in **a** and **b**) are also labelled. All bathymetry data is sourced from GEBCO^81^.

**Fig. 2:**
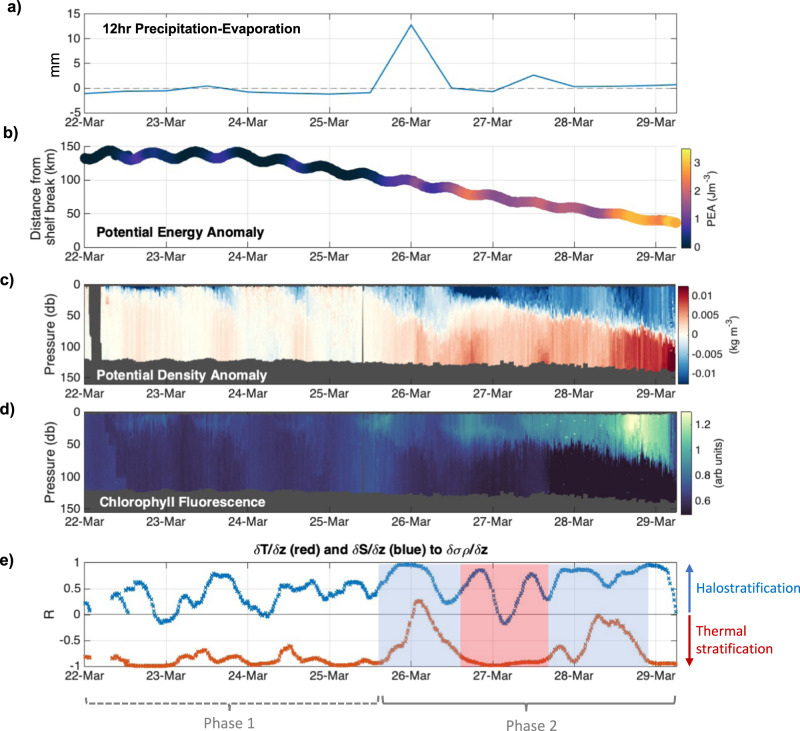
The onset of seasonal stratification. **a** Total cumulative 12-h precipitation-evaporation (mm; ERA-Interim^38^) with positive values indicating that precipitation > evaporation; **b** The distance (in km) the glider was from the shelf break, with the colours indicating the potential energy anomaly (Jm^−3^); **c** The potential density anomaly (*σρ*; kg m^−3^) observed by the glider; **d** Observed chlorophyll fluorescence (arb units); **e** Correlative analysis to determine if the change in potential density (∂*σρ*/∂z) with depth is attributable to changes in salinity (∂S/∂z, blue) or temperature (∂T/∂z, red) gradient. Phase 1 refers to the period before stratification onset, and Phase 2 is after stratification has been triggered. The shaded areas are to highlight which part of the stratification observed in Phase 2 is controlled by salinity (blue) and temperature (red). The glider moved into the shelf break on the 29th March and returned on shelf on the 2nd April. For clarity this has been removed from the figure, but an on-shelf mooring confirmed that stratification was sustained over this time (see Wihsgott et al.^17^).

An ocean glider following a repeat transect in the Celtic Sea (Fig. 1) provided measurements that captured the onset of seasonal stratification (see Methods). The daily heating and cooling cycle was identified between the 22nd and 25th March (labelled as Phase 1 in Fig. 2), demonstrated by daytime increases in the water column potential energy anomaly ($\phi$, J m^−3^; Fig. 2b), which refers to the amount of mechanical energy (per unit depth) required to mix the water column^26,27^ (see Eq. (1)). The strength of stratification is proportional to *ϕ*, with the water column being homogenous when *ϕ* is equal to zero:

1 $${{{{{\rm{\phi }}}}}}=\frac{1}{h}{\int }_{h}^{0}(\hat{\rho }-\rho )\,{gz}\,{dz}\, \qquad \hat{\rho }=\frac{1}{h}{\int }_{h}^{0}\rho\, {dz}.$$

Where $\rho$ (*z*) is the density profile (kg m^−3^) over a water column of depth *h* (m), and $\hat{\rho }$ is the water column mean density (kg m^−3^).

However, following a rain event on the evening of the 25th March, near-surface potential density, *σ*_*θ*_ (kg m^−3^) decreased by 0.006 kg m^−3^ over a 12-hour period, resulting in sustained stratification, i.e. *ϕ* > 0 Jm^−3^ (labelled as Phase 2 in Fig. 2). We conclude that this stratification was due to the addition of freshwater and not due to thermal conditioning, since observed night-time (21:00 to 03:00) changes in the potential density gradient ∂*σ*_*θ*_/∂z (see Fig. S1) are strongly correlated to changes in the vertical salinity gradient, ∂S/∂z (*r* = 0.9) and poorly correlated with the vertical temperature gradient, ∂T/∂z (*r* = 0.1; *p* = <0.001). Interestingly, the weakly positive correlation for ∂T/∂z indicates a cold-water cap, that works to reduce buoyancy and would promote convective overturning without the stabilising effect of the observed rain-induced freshwater stratification. Satellite-derived estimates for precipitation show that this rain event between the 25th and 26th March covered the majority of the Celtic Sea region (see Fig. S2).

This layer of freshened water was largely isolated from the stirring influence of tidally-driven turbulence generated at the seabed, and thus retained some of the standing phytoplankton stock. This is demonstrated by an increase in near-surface chlorophyll fluorescence (Fig. 2d) immediately following the initial onset of rain-induced stratification. Maximum chlorophyll fluorescence occurred four days after the observed rain event, on the 28th March (Fig. 2d). Enhanced optical backscatter recorded by the glider (Fig. S3) in the surface layer provided further confidence that this increase in chlorophyll fluorescence was due to increased phytoplankton biomass rather than the result of photo-acclimation^28–30^. Analysis of samples collected during process cruises soon after the glider deployment confirmed that elevated chlorophyll fluorescence continued beyond the 2nd April and throughout the spring period^31,32^. The timing of seasonal stratification observed in this study was consistent with conditions observed up to 120 km from the shelf break^17^, confirming that these results are representative over regional scales.

Sustained stratification (*ϕ* > 0 Jm^−3^) will only occur when the net daily buoyancy contribution is positive, managing to outcompete the combined mixing effects of wind and tides (see Eq. [1] and “Methods” section), and night-time periods when convection is likely. Storm activity is considered to delay the onset of stratification on the NW European shelf due to increased wind mixing^9^, while the relative phase of the spring-neap cycle contributes substantially to the timing of stratification through local variability in tidal mixing^9,15,16^. Changes in potential energy anomaly can be partitioned as follows:

2 $$\underbrace{\frac{d{\phi }}{dt}=\frac{d{\phi }_{heat}}{dt}+\frac{d{\phi }_{{rain}/{evap}}}{dt}}_{{{{{{\mathrm{Contributions}}}}}} \, {{{{{\mathrm{to}}}}}} \, {{{{{\mathrm{buoyancy}}}}}} \, ( {{{{{\mathrm{can}}}}}} \, {{{{{\mathrm{be}}}}}} \, {{{{{\mathrm{positive}}}}}} \, {{{{{\mathrm{or}}}}}} \, {{{{{\mathrm{negative}}}}}})}-\underbrace{\frac{d{\phi }_{wind}}{dt}-\frac{d{\phi }_{tides}}{dt}}_{{{{{{\mathrm{Contributions}}}}}} \,{{{{{\mathrm{to}}}}}} \, {{{{{\mathrm{mixing}}}}}}}$$

**Fig. 3:**
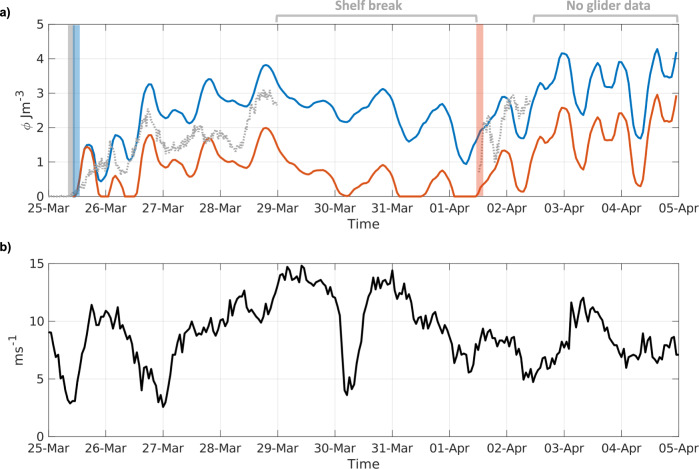
Testing the physical controls on stratification onset. **a** The potential energy anomaly (*ϕ*) calculated with (blue line) and without (red line) the buoyancy from rain and evaporation, compared to the observed potential energy anomaly (Jm^−3^; dotted grey line) from the glider. Note that, unlike the glider, the modelled *ϕ* assumes a fixed point (the closest location to the Central Celtic Sea, CCS, site) and is thus independent of processes observed by the glider as it moved towards the shelf break. As such, all data recorded when the glider moved into the shelf break regime have been omitted, and there is no glider data after the 2nd April. The coloured bars are indicative of when sustained seasonal stratification began; **b** The observed 10 m wind speed recorded from the ODAS Met Buoy (in ms^−1^; see “Methods” section) at the CCS site.

Analysing the separate contributions to *ϕ* (Eq. [2] and “Methods” section), allows a quantitative assessment of the importance of rainfall in triggering stratification. To compare the relative magnitude of the different contributors to buoyancy, time series of *ϕ* were calculated (see “Methods” section) with and without the influence of the rain and evaporation (Fig. 3a). Results show that increased freshwater buoyancy on the 25^th^ March 2015 was sufficient to allow sustained stratification to form a week earlier than predicted when only considering thermal inputs. While weak stratification would have likely developed on the 26th March by thermal inputs alone, there was inefficient net buoyancy input to outcompete increased mixing by wind (Fig. 3b). Ultimately, the water column would have homogenised on the 30th March if this were the case (Fig. 3a), with sustained stratification later developing on the 1st April, 7 days later than the observations. In addition to the freshwater buoyancy effects of rainfall, there was also an associated positive thermal buoyancy input of up to 0.35 Wm^−2^ (Fig. S4) from the sensible heat (known as precipitation-induced sensible heat) transferred into the ocean by rainfall^33,34^. As this only accounted for <1% of the maximum daytime heat flux into the ocean, it is not considered to be a controlling factor.

To determine whether the observed rain event contributed to the observed surface freshening, we can estimate the salinity change in the surface mixed layer ($\triangle S$) using:

3 $$\triangle S={S}_{o}\left(1-\frac{{Z}_{{SML}}}{{Z}_{{SML}}+P}\right).$$

Where *So* is the initial salinity in the surface mixed layer, *Z*_*SML*_ is the depth of the surface mixed layer (m), and *P* is the rain depth (m).

**Fig. 4:**
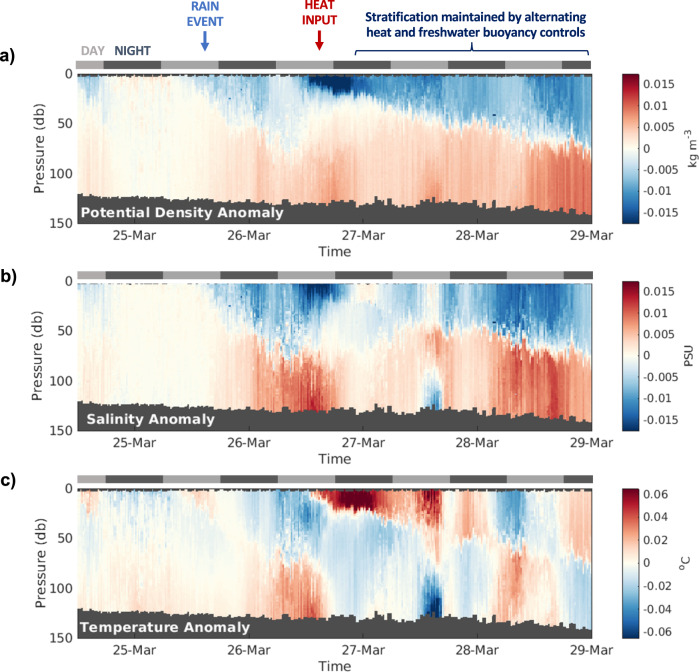
Transects at the onset of stratification. Transects recorded by the glider between the 24th and 29th March that show the anomaly (relative to the mean profile) for **a** potential density (kg m^−3^), **b** salinity (PSU) and **c** temperature (^o^C). A scale has been included to indicate night and day cycles, as well as labels to indicate the rain event and heat input.

From 18:00 on the 25th March to 06:00 on the 26th March, 13.1 mm of rainfall coincided with a surface freshening of Δ*S* = 0.0124. Taking an average *Z*_*SML*_ of 44 m over the same 12-h time period, the estimated salinity change in the surface mixed layer was calculated at Δ*S* = 0.0104, which confirms that the observed change in surface salinity can be largely attributed to the salinity dilution by rainfall. While the remaining freshwater could be due to uncertainties in the ERA-Interim precipitation data, the sustained winds of ~10.3 ms^−1^ during the same time implies surface wind-driven transport^35^. A second, smaller (2.8 mm) rain event occurred on the 27th March 2015 (Fig. 2), and accounted for over half of the observed freshening in the surface layer. The remaining contribution to surface layer freshening could also have been advected into the region due to wind-driven transport, which dominated the stratification control up until the relaxation of westerly winds from the 5th April^35^, by which point thermal heating began to dominate. For the purposes of this paper, we focus only on the buoyancy contributions from rain, summarised in Fig. 4, whereby a rain event reduced the surface density (Fig. 4a) via a decrease in the surface salinity (Fig. 4b) and triggered stratification, which was later strengthened by surface heating (as seen by the temperature anomaly in Fig. 4c). Seasonal stratification was further maintained through episodic mixing events, such as through the wind event on the 29th March (as seen in Fig. 3b), by alternating thermal and freshwater buoyancy controls (Fig. 4).

**Fig. 5:**
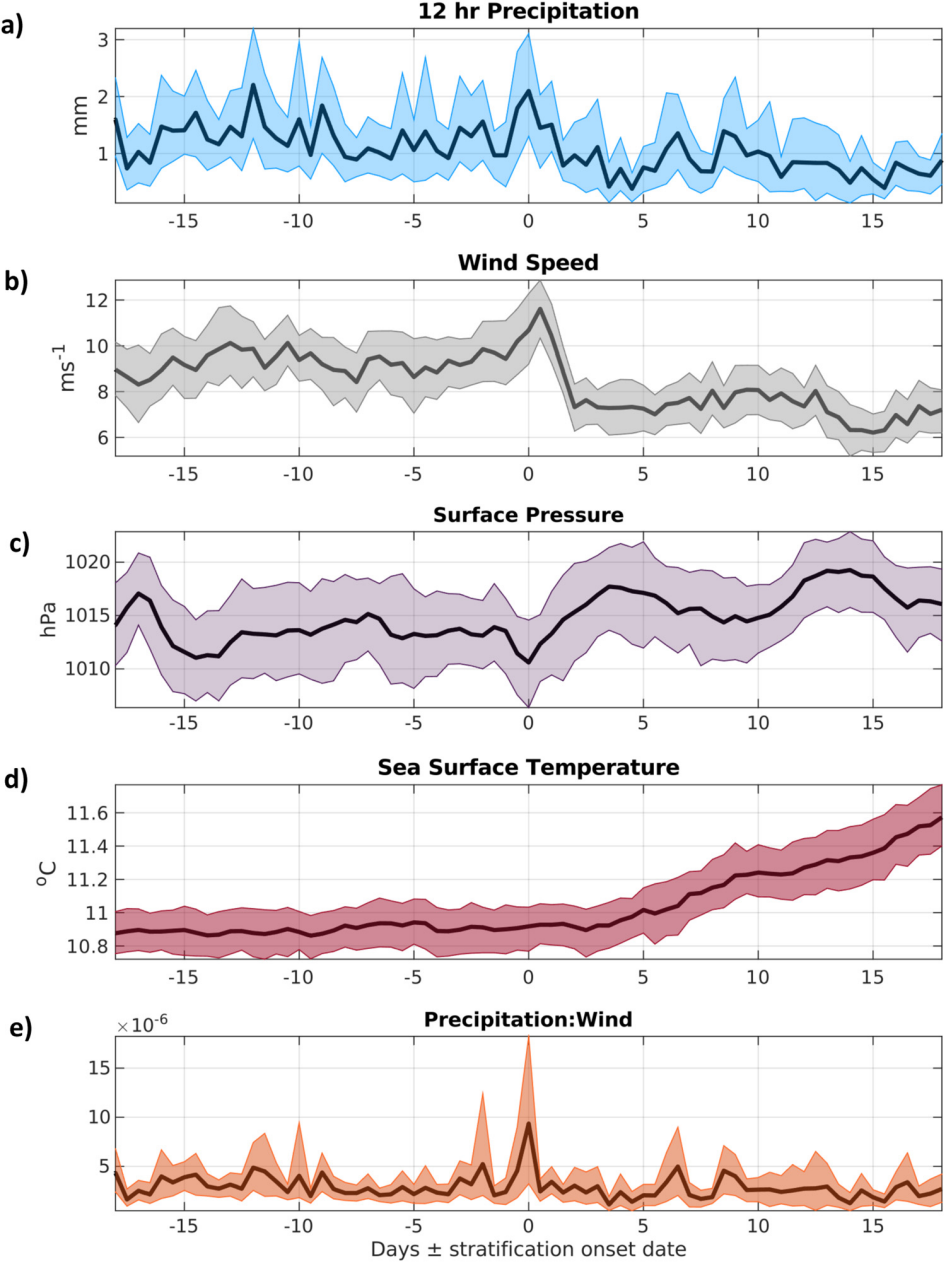
Meteorological conditions at the onset of stratification. Averages of meteorological parameters (ERA-Interim^38^) from 1982 to 2015; **a** 12-h precipitation (mm); **b** wind speed at 10 m; **c** sea surface pressure (hPa); **d** sea surface temperature (°C), all relative to the stratification onset date; and **e** the rain-to-wind ratio (dimensionless). The shaded areas are the 95% confidence limits, calculated using the bootstrap percentile method.

The influence of climate change on seasonal stratification is still under debate^36^. While regional warming may result in an earlier onset of stratification^37^, more energetic winds may also act to delay it^9^. Therefore, to test the repeatability of the physical processes discussed here on the formation of seasonal stratification, mean averages of rainfall, wind speed, mean sea level pressure and sea surface temperature (ERA-Interim^38^) were compared to the predicted onset dates of seasonal stratification in the Celtic Sea from 1982 to 2015 (Fig. 5) using model results from a hydrodynamic model, NEMO^39,40^ (see “Methods” section).

Rainfall (Fig. 5a) and low-pressure (relative to the mean air pressure 20 days ± the stratification onset date; Fig. 5c) simultaneously occurred at the same time on or shortly before the modelled stratification onset in 30 out of 34 years (88% of the time), suggesting March 2015 was not a unique coincidence, but reflects a typical conditioning scenario. Furthermore, wind speeds typically peaked after rainfall (Fig. 5b), which promotes wind-driven transport of freshwater that could further strengthen the existing halo-stratification^35^. Sea surface temperatures typically increased 2 to 3 days after the initial density stratification onset (Fig. 5d) and coincided with a period of meteorological quiescence after the storm had passed. This supports the proposed concept that seasonal stratification is often triggered initially by halo-stratification rather than being thermally induced, and challenges how we currently understand the role of spring storms in controlling the timing of seasonal stratification in temperate shelf seas.

**Fig. 6:**
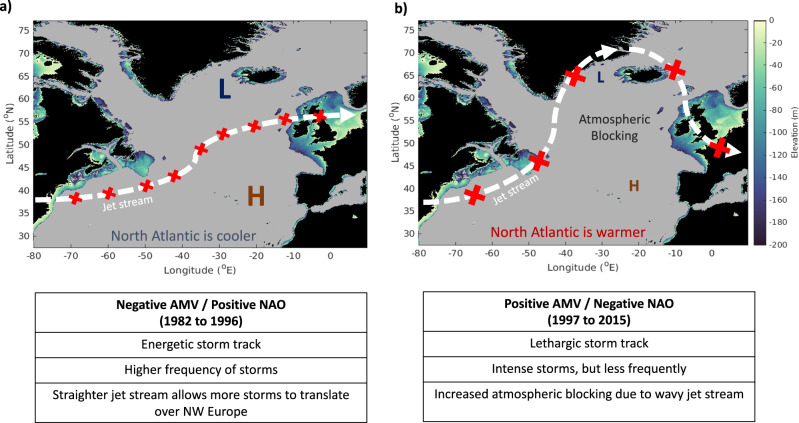
The jet stream across the North Atlantic. Schematics, modified from Jardine et al.^55^, detailing the changes of the North Atlantic Storm Track with respect to the Atlantic Multidecadal Variability (AMV) and the North Atlantic Oscillation (NAO), where **a** denotes a negative (positive) AMV (NAO) from 1982 to 1996, and **b** denotes a positive (negative) AMV (NAO) from 1998 to 2015. The table in the lower plots summarises the key changes in storm track characteristics over Northern Europe. The coloured sections represent the bathymetry of the continental shelf seas, defined as <200 m deep. Red crosses on the jet stream (white dashed arrow) are indicative of storms; larger crosses are more energetic storms. All bathymetry data is sourced from GEBCO^81^.

However, an added complexity is that regional meteorological conditions across NW Europe are highly dependent on large-scale climate-oscillations in the North Atlantic, such as the Atlantic Multidecadal Variability (AMV) and the North Atlantic Oscillation (NAO). The AMV is defined as the long-term temperature anomaly in the North Atlantic^41^. The AMV was generally considered to be in a negative phase (relatively cool) from 1960 before transitioning into a generally positive phase (relatively warm) in the mid-1990s, which remained until our 2015 study period. This phase shift in the AMV roughly coincides with that of the NAO, which is defined as the relative strength of the Azores-Icelandic sea level pressure gradient^42,43^. The NAO was often positive from 1965 to 1995^44^, resulting in a straight jet stream across the North Atlantic (Fig. 6a) that brought stormier, wetter conditions over NW Europe^45,46^. From the mid-1990s, the NAO has been in a predominantly negative phase^47,48^ with a consequently less stable (wavy) jet stream (Fig. 6b).

**Fig. 7:**
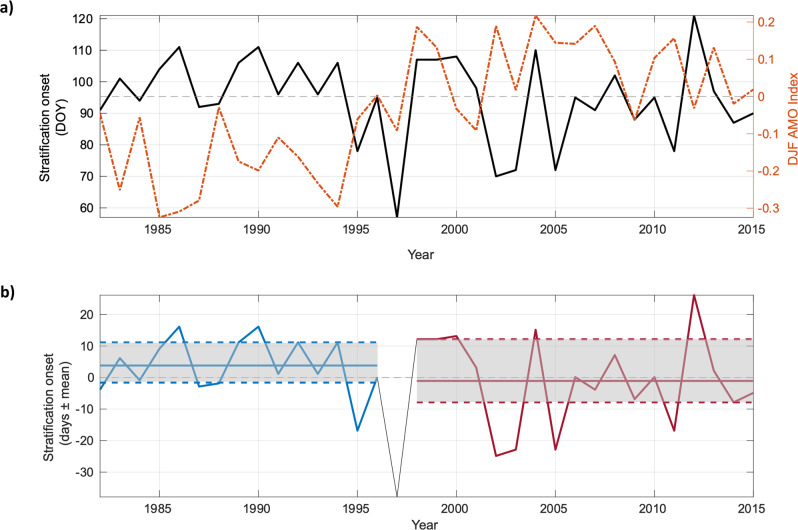
Stratification onset dates compared to the Atlantic Multidecadal Variability (AMV). **a** Comparison of the stratification onset dates (solid black) to the December-January-February (DJF) AMV Index (dashed red) from 1982–2015; **b** The stratification onset dates in days ± from the mean of the full period, from a negative (blue) to positive (red) AMV phase; the mean (solid coloured lines), as well as the 25th and 75th percentiles (dotted coloured lines), are also marked and shaded grey. Note that 1997 is an anomalous year that occurs directly on the transition period between AMV phases and has such been omitted from the phase statistics. The AMV Index is the smoothed timeseries^82^, accessed here: https://psl.noaa.gov/data/timeseries/AMO/.

Although the model simulation (duration 34 years) does not cover a whole AMV cycle (60-80 years), it does include periods of positive and negative AMV. Comparing the modelled onset dates of stratification to the AMV phase (Fig. 7) reveals a distinct shift in the year-to-year variability in stratification onset date that coincides with a phase shift in the AMV. Stratification onset dates exhibited an almost two-fold increase in variability (the difference in the 25th and 75th percentiles) during the positive AMV phase relative to when the AMV was in a negative phase: equivalent to a range of 20 days and 13 days respectively. Comparisons of stratification onset to positive and negative NAO phases were more ambiguous (see Figs. S5–S7), however given that the NAO is the dominant control of climate variability in the North Atlantic^44,45,49^, and previous studies have shown a positive correlation between the NAO phase and regional timing of stratification^9^, it cannot be discounted as a further control on stratification variability in the region.

While we have not been able to extrapolate these results across multiple AMV cycles, the abrupt step change in stratification onset dates in relation to the AMV suggests that changes in large-scale atmospheric conditions, such as those resulting from a phase-shift from a negative to positive AMV, exhibit a first-order control on the onset of stratification during this time. The negative AMV phase from 1982 to 1996 likely promoted a more energetic storm track with stronger westerly winds^43^ and a higher frequency of intense cyclones^50,51^. When combined with a straighter jet stream from a predominantly positive NAO, this would result in more regular and prolonged periods of high wind stress, resulting in a bombardment of storms and associated high winds that would act to prevent sustained stratification and homogenise the water column, thus delaying the onset of seasonal stratification. This is supported by the relatively stable period of stratification onset dates that occurred throughout the negative AMV phase (Fig. 7).

Conversely, from 1998 to 2015, the combined effects of a warmer North Atlantic (positive AMV) and a wavy jet stream (negative NAO) would promote a lower frequency of intense storms being delivered to NW Europe. Conditions for storm genesis are unfavourable during positive AMV periods^52^ and, combined with a negative NAO, this results in a wavier jet stream and increases the likelihood of atmospheric blocking over NW Europe^53,54^, which deflects migratory cyclones and promotes periods of relatively low storm activity (Fig. 6b).

Once initial stratification had been triggered by a storm event the relatively quiescent conditions between these less frequent storm systems (during positive AMV and negative NAO periods) would promote continual strengthening of stratification, with homogenisation of the water column only then likely to occur during extreme conditions, such as from an explosive cyclone^50,52^. This could explain the modelled conditions in 2012 when an initial period of sustained stratification formed on the 19^th^ March, producing a peak in *ϕ* of 23.3 J m^−3^ on the 31st March (see Fig. S8) and promoted significant phytoplankton growth^55^. This stratification was subsequently eroded following a week-long period of high wind speeds. Sustained stratification was again re-established on the 30th April 2012, following a week-long period of heavy rain and sustained high wind speeds. This was the latest predicted seasonal stratification onset date identified across the 34-year analysis period.

Here, we have demonstrated that the onset of seasonal stratification is partially dependent upon the timing and intensity of spring storms and their associated precipitation. Storms that bring a lot of rain have a higher potential to trigger stratification, provided the enhanced freshwater buoyancy is sufficient to outcompete mixing by convection and wind and tidal stresses at the time of initiation. This rain-induced stratification will continue to strengthen throughout the spring period unless the mixing effects from subsequent storms are sufficient enough to reduce *ϕ* to 0 Jm^−3^ or below. An extreme example of this occurred in 1997 (identified as an anomalous year in Fig. 7b), where relatively calm conditions followed a period of high wind and rain and resulted in sustained stratification forming on the 26th February (see Fig. S9). As no subsequent storms occurred to erode the stratification through wind mixing, it continued to strengthen, and this region ultimately experienced an unusually early stratification onset date. Jardine et al.^55^ describe the biogeochemical response of phytoplankton to such an early stratification onset date, with phytoplankton growth in 1997 occurring 3 weeks after the onset of seasonal stratification as seasonal light levels were insufficient to initiate growth despite stratification trapping phytoplankton cells in the upper water column.

## Discussion

How the frequency and intensity of storms across NW Europe will change in the future is still uncertain. Some studies predict that storms and other high-wind events will increase across much of Europe^56,57^, whereas others predict a reduction in storm activity^58–60^. Despite this uncertainty, our study provides insight into the potential impacts of storm variability on critical stages of shelf sea seasonal cycles and prompts further debate on how the intensity and character of changing storm activity will impact regional seas. Strong winds associated with storms will act to mix the water column and delay stratification, whereas the increased surface buoyancy from storm-induced rainfall may act to stabilise the water column, triggering stratification much earlier than when considering thermal buoyancy alone. The onset of sustained seasonal stratification is thus dependent on the strength, duration and track of storms, and their rain-to-wind ratios.

It is uncertain whether future predicted conditions will lead to less variability in seasonal stratification onset due to the high wind stress associated with changing storm activity, or whether increased intense rainfall associated with an accelerated hydrological cycle^61^ under future climate conditions will lead to a more variable stratification onset. For example, the area affected by extreme winds from extratropical cyclones is predicted to broaden by as much as 40% by the end of the century^60^, which could potentially delay the onset of stratification, however rainfall from extratropical cyclones is also expected to increase by as much as 50% in future climates^62^, which may balance or overcome the mixing from winds and trigger stratification. Some regional ocean model studies have shown stratification occurring earlier under future climate conditions^36^, and this may mean an increased importance of rainfall events in driving the onset of seasonal stratification, as it coincides more with winter storms.

While we have focused on the NW European shelf, the interactions between high precipitation events and winter seas highlighted in this study are relevant on a global scale. Several continental shelf seas experience heavy rain or storm activity, particularly those beneath so-called atmospheric rivers:^63,64^ jets of water vapour that typically occur ahead of extratropical cyclones^65,66^. Equivalent in freshwater volume flux to the world’s largest rivers^63,66^, atmospheric rivers occur in both northern and southern hemispheres, including NW Europe, the Western United States, and Southeast America^63,66,67^, and have the potential to deliver large quantities of rain to the continental shelf sea regions. For example, extreme flooding events in the UK have been linked to atmospheric rivers^65,68^, and Blamey et al.^69^ found that 70% of the top 50 winter rainfall extremes across the west coast of South Africa were also linked to atmospheric rivers, which could potentially influence the seasonal progression of shelf sea physics. As the frequency and severity of atmospheric rivers are expected to increase in future climate scenarios^65,70,71^, it can be assumed that high precipitation events will more prominently influence the physical structure of shelf sea environments across the globe in the future.

Rainfall from intense extratropical cyclones is expected to increase at a rate of 7% per K^72^, and by as much as ~50% with a 4 K warming^62^, which increases the potential of rain-induced stratification as the climate warms. Furthermore, the intensity and distribution of intense extratropical cyclones in the northern hemisphere is expected to change in warmer climates, with a reduction in the overall number of extratropical cyclones^60^ but a predicted 5% increase in extreme cyclones^60,62^. This is analogous to the positive AMV/negative NAO conditions in Fig. 6b, where storms are stronger but less frequent, and could suggest stratification onset dates will become more variable in the future. This is in contrast to previous studies that exclude rainfall as a trigger mechanism for stratification;^9,24,73^ and has profound ecological implications for the spring phytoplankton bloom initiation and associated trophic dependencies^55^.

In this study, high resolution spatial and temporal data collected from an autonomous underwater glider allowed further insight into the controlling physical drivers of seasonal stratification in the Celtic Sea. Expanding the study using a 3D model, the decadal variability of shelf sea stratification on the NW European Shelf was linked to large-scale climatic variability in the North Atlantic. Future work needs to further investigate the implications of passing storm systems on productivity and ecosystem function from climatic variability, particularly on centennial timescales. We emphasise that rainfall from passing storm systems cannot be ignored as an initial trigger for seasonal stratification in temperate regions.

## Methods

Data used in this study was from one of four gliders deployed from November 2014 to August 2015 as part of the UK NERC-DEFRA Shelf Sea Biogeochemistry Programme (2014 - 2019). The gliders were programmed to do repeated transects between the Central Celtic Sea site (49^o^ 24.00 N, 8^o^ 36.00 W) and the shelf break site (CS2; 48^o^ 34.26 N, 9^o^ 30.58 W). Following deployment, the glider data was processed using a Matlab-based Glider Toolbox that uses a flight model by Merckelbach et al.^74^ and a thermal inertia correction based on Leuck and Picklo^75^ and Garau et al.^76^. Only data collected from one of the four SSB gliders were used in this study (Unit 419, “Fortyniner” deployed from the 22 March 2015 to the 2 April 2015).

The glider was fitted with a pumped seabird CTD package to measure pressure, temperature and conductivity, and a Wet Labs triplet puck to measure chlorophyll-a fluorescence, backscatter and coloured dissolved organic matter (CDOM). Unit 419 travelled a total of 315 km before recovery and made one complete transect between the CCS (Mooring) and CS2 (shelf break) sites. As Unit 419 was deployed in late March, thermal inertia was minimal, and no visible drift in the data was detected. Due to the scarcity of comparable CTD casts, the glider data from Unit 419 only had one round of calibration offset calculations.

Full details of the glider deployments and the data processing can be found in the Data Report: https://www.bodc.ac.uk/data/published_data_library/catalogue/10.5285/dd2a4f57-5943-68c7-e053-6c86abc0eb55/.

Observational meteorological data was recorded by the UK Met Office Ocean Data Acquisition Sensor (ODAS) Buoy, which measured wind speed (ms^−1^), air density (kg m^−3^), mean surface sea level pressure (hPA) and relative humidity (%). These variables were used to calculate the 2015 surface heat fluxes (Wihsgott et al.^17^ and section 5.3) for use in the idealised potential energy anomaly calculations in Fig. 3.

A measure of stratification in shelf seas is the potential energy anomaly, *ϕ* (Jm^−3^, Simpson and Bowers, 1981), defined as the amount of mechanical energy needed to mix the water column. As such, the strength of stratification is proportional to *ϕ*, with the water column being homogenous when *ϕ* is equal to zero:

4 $$\phi=\frac{1}{h}{\int }_{h}^{0}(\hat{\rho }-\rho )\,{gz}\,{dz}\, \qquad \hat{\rho }=\frac{1}{h}{\int }_{h}^{0}{dz}.$$

Where *ρ* (*z*) is the density profile (kg m^−3^) over a water column of depth *h* (m), and $\hat{\rho }$ is the water column mean density (kg m^−3^).

The change in potential energy anomaly (*ϕ*) with time (t) can be subdivided into its separate components, including heating/cooling, rain/evaporation, wind mixing and tidal mixing.

5 $$\frac{d{\phi }}{{dt}}=\frac{d{\phi }_{{{\mbox{heat}}}}}{{dt}}+\frac{d{\phi }_{{{\mbox{rain}}}/{{\mbox{evap}}}}}{{dt}}-\frac{d{\phi }_{{{\mbox{wind}}}}}{{dt}}-\frac{d{\phi }_{{{\mbox{tides}}}}}{{dt}}\quad\left({{{{{\rm{W}}}}}}{{{{{{\rm{m}}}}}}}^{-3}\right)$$

Heating and cooling (*ϕ*_*heat*_) influences buoyancy by:

6 $$\frac{d{\phi }_{{{\mbox{heat}}}}}{{dt}}=\frac{{ag}{Q}_{{{\mbox{net}}}}}{2{C}_{p}}\quad\left({{{{{\rm{W}}}}}}{{{{{{\rm{m}}}}}}}^{-3}\right)$$

Where *Q*_*net*_ is the total heat flux, $a$ is the expansion coefficient due to temperature, $g$ is the acceleration due to gravity and *C*_*p*_ is the specific heat capacity of seawater.

However, freshwater input from rain also has a positive buoyancy effect. By combining the buoyancy effects from both thermal heating and freshwater from rain events, then:

7 $$\frac{d{\phi }_{{heat}}}{{dt}}+\frac{d{\phi }_{{rain}/{evap}}}{{dt}}=\frac{d{\phi }^{b}}{{dt}}=\frac{g}{2}\left(\frac{a{Q}_{{net}}}{{C}_{p}}+\dot{P}\triangle \rho \right)\quad\left({{{{{\rm{W}}}}}}{{{{{{\rm{m}}}}}}}^{-3}\right).$$

Where $\dot{P}$ is precipitation rate (m s^−1^) and $\triangle \rho$ is the density difference between seawater and freshwater.

The mixing terms, from wind (${\phi }_{{wind}}$) and tidal mixing (${\phi }_{{tides}}$), can be calculated by Eqs. (8) and (9):

8 $${\frac{d{\phi }_{{wind}}}{{dt}}=\epsilon }_{1}{k}_{s}{\rho }_{s}\frac{{W}^{3}}{h}\quad\left({{{{{\rm{W}}}}}}{{{{{{\rm{m}}}}}}}^{-3}\right).$$

9 $$\frac{d{\phi }_{{tides}}}{{dt}}=\frac{4}{3\pi }{\epsilon }_{2}{k}_{b}\rho \frac{{u}_{1}^{3}}{h}\quad\left({{{{{\rm{W}}}}}}{{{{{{\rm{m}}}}}}}^{-3}\right).$$

Where ${\epsilon }_{1}$ and ${\epsilon }_{2}$ are the mixing efficiencies for winds (0.023) and tides (0.004) respectively, *u*_*1*_ is the tidal stream amplitude, $\rho$ and ${\rho }_{s}$ are the densities of seawater and air and *k*_*b*_ is the bottom drag coefficient (0.0025). The term *k*_*s*_ is taken to be *C*_*D*_*γ*_*s*_, where *C*_*D*_ is the drag coefficient (0.0012) and *γ*_*s*_ is the slippage factor (0.02).

The model used in this study is the 7 km Atlantic Margin Model (AMM7) configuration of NEMO (Madec, 2015), covering the NW European Shelf and part of the Eastern Atlantic (20^o^W, 40^o^N, 13^o^E, 65^o^N), with a resolution from 9.4 to 5.2 km (averaging a 7.4 km mean resolution). Bathymetry is derived from the Northwest European Shelf Operational Oceanographic System (NOOS) and uses a hybrid z-s coordinate system with 51 vertical levels. Vertical levels are uniform near the ocean surface across the whole domain, allowing for more consistent ocean-air exchanges. Initial conditions and boundary forcing were sourced from the 1/4° ORCA025 hindcast of GO5.0 (Megann et al.^77^ more details of GO5.0’s initialisation can be found in Ingleby and Huddleston^78^). Meteorological and atmospheric forcing were sourced from ERA-Interim^38^. Following an initial spin-up period from 1981, the model ran continuously from 1982 to 2015.

A full review of the AMM7 sensitivity tests and validations with observations and previous models (e.g., POLCOMS) can be found in O’Dea et al. (2017)^38^. Furthermore, Luneva et al.^79^ found that biases between the model setup and observations were <0.05^o^C at the surface and <−0.01 °C across the whole domain. The onset date for seasonal stratification in the model has also been validated against the glider observations (Fig. S10) and confirms a 3-hour offset in stratification timing, likely due to the model resolution ( ~ 7 km) or discrepancies between the observed and modelled (ERA-Interim) precipitation data.

As the model used ERA-Interim atmospheric forcing, the decision was made to continue using ERA-Interim for further analyses in this study, despite the improved ERA5 release. ERA5 precipitation has been shown to have lower bias over the mid-latitude regions than ERA-Interim^80^, however this was deemed a suitable compromise in order to maintain consistency.

## Supplementary information

Supplementary Information

## Data Availability

The AMM7 model data used NEMO V3.6_stable, available from: https://forge.ipsl.jussieu.fr/nemo, and the biogeochemical model ERSEM, available here: https://github.com/pmlmodelling/ersem/. Model data is stored on the Jasmin supercomputer, here: /gws/nopw/j04/ssb/data/internal/AMM7-hindcasts/v0.2repr/. Access to this directory can be requested from Yuri Artioli (yuti@pml.ac.uk) at the Plymouth Marine Laboratory. The glider data is available through the British Oceanographic Data Centre (BODC) available from: https://www.bodc.ac.uk/data/published_data_library/catalogue/10.5285/dd2a4f57-5943-68c7-e053-6c86abc0eb55/. Meteorological data collected by the ODAS Met Buoy is managed by the UK Met Office and is available on request. ERA-Interim data is available to download from the European Centre for Medium-Range Weather Forecasts (ECMWF).

## Code availability

The software package Matlab was used for all data analysis and production of figures; these codes are available upon request from the lead author.
