# Supplementary material for: Rain triggers seasonal stratification in a temperate shelf sea
Source: Nat Commun. 2023 Jun 2;14:3182. doi: 10.1038/s41467-023-38599-y (PMC10238390; doi:10.1038/s41467-023-38599-y)
Supplement: Supplementary file 1 — Supplementary Information [file 41467_2023_38599_MOESM1_ESM.pdf]

Supplementary Figures

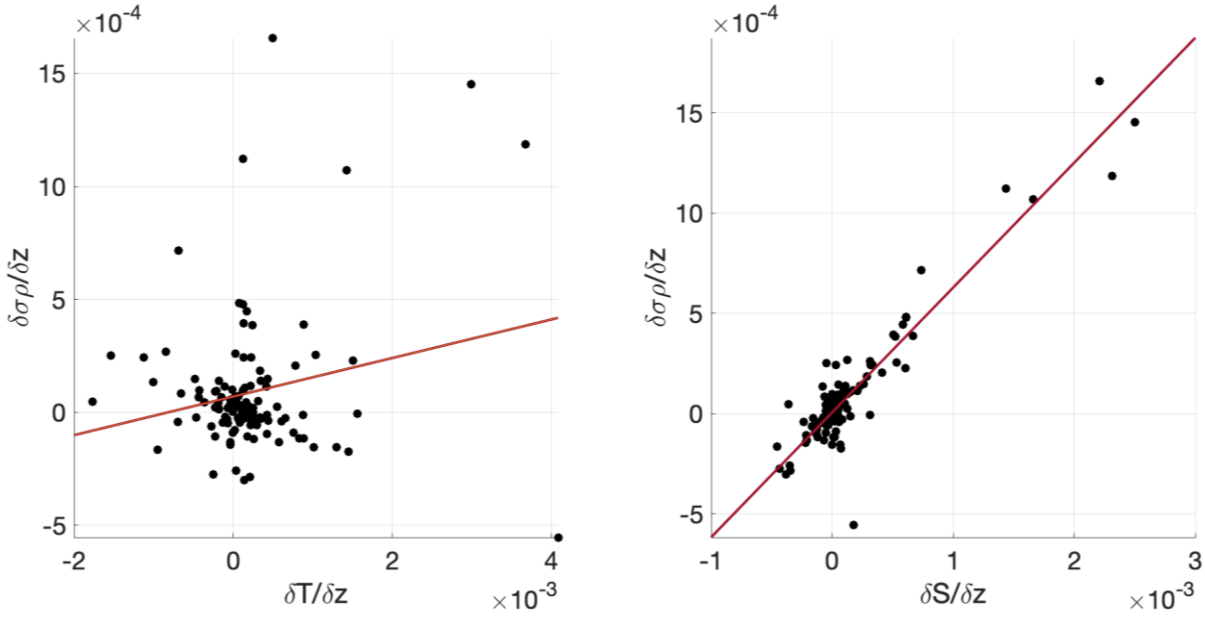

Figure S1: Temperature and salinity correlation to potential density. Comparisons between the changes in temperature and salinity (right) with depth to the changes in the potential density with depth, between 21:00 and 03:00 on the 25th and 26th March 2015.

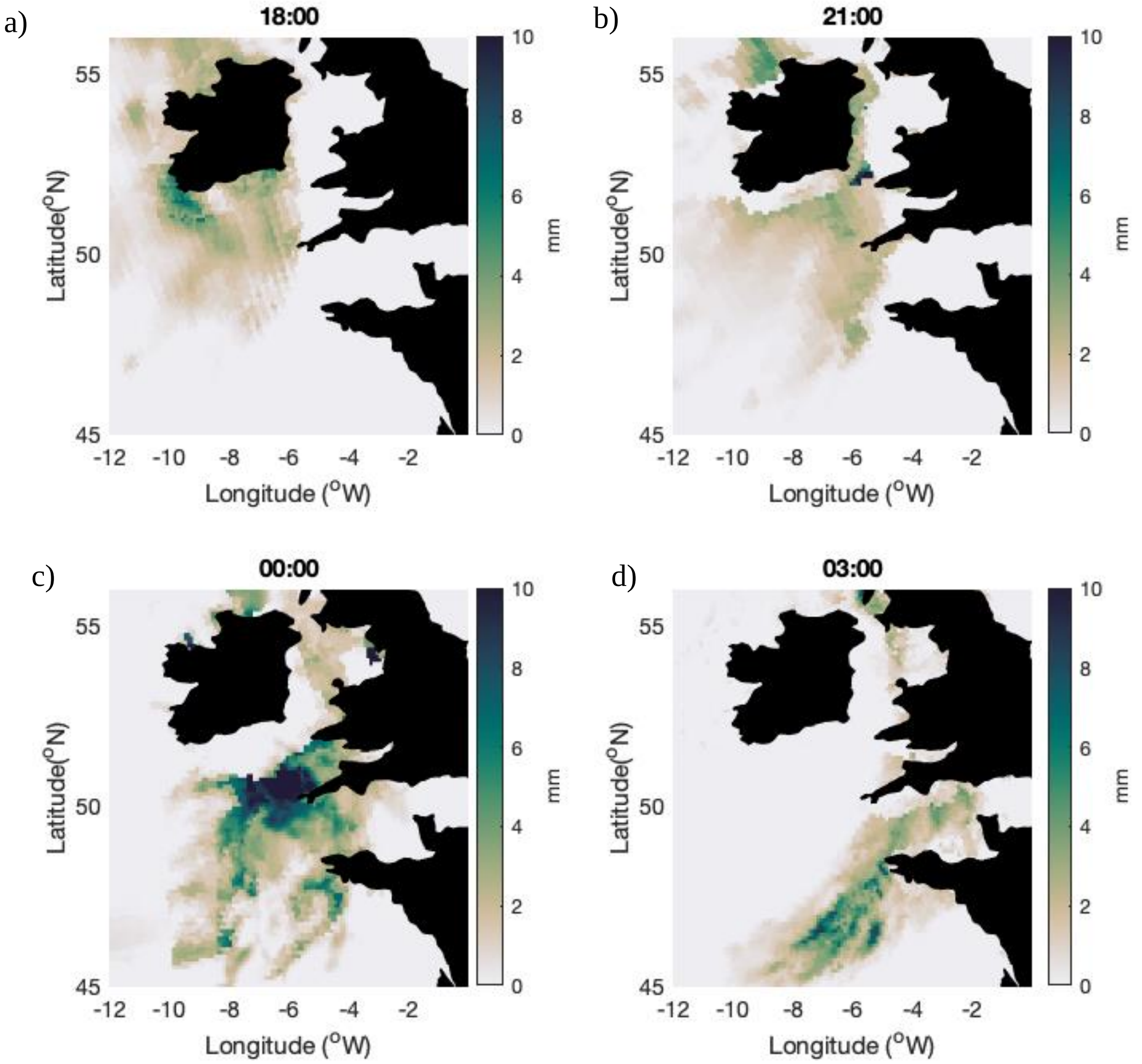

Figure S2: Satellite-derived precipitation (IMERG) over the Celtic Sea at 3 hour intervals. a) 18:00; b) 21:00; c) 00:00 and d) 03:00 from the 25th to the 26th March 2015, to show the spatial distribution of the precipitation over the Celtic Sea. Precipitation satellite data is the half hourly 0.1x0.1degree IMERG product (Huffman et al, 2019). Accessed: 2019.07.13.

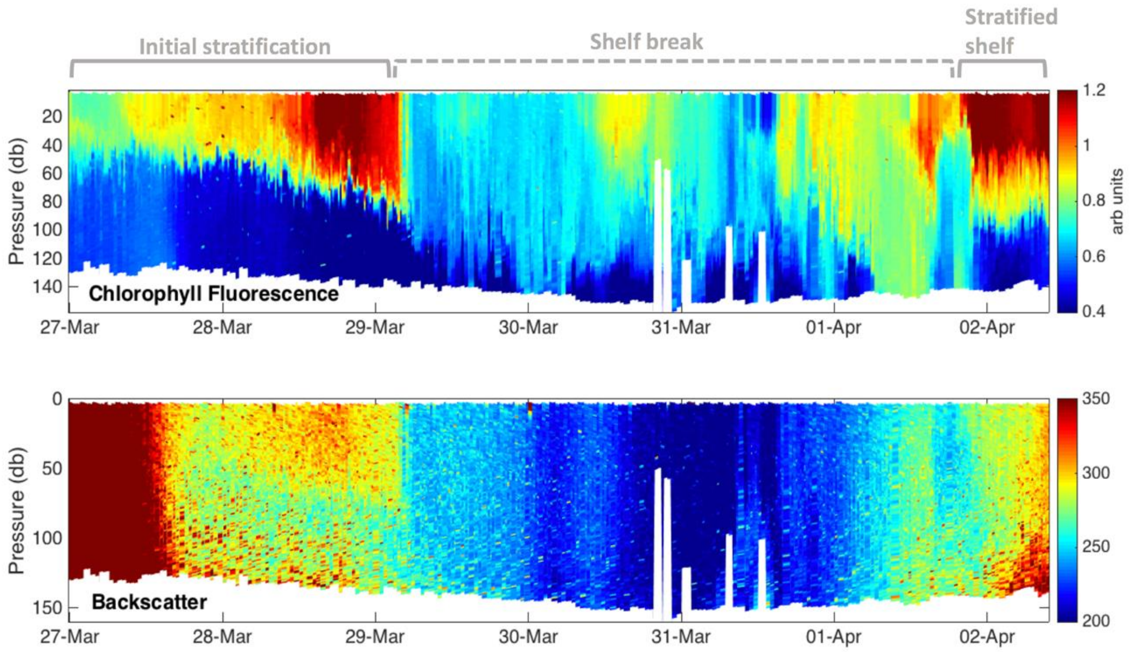

Figure S3: Chlorophyll-a fluorescence vs optical backscatter. Comparison between chlorophyll fluorescence (upper) and backscatter (lower), measured by the glider, are following the rainfall event on the 25th March 2015. Note, this transect only includes the period between the 27th March and the 2nd April 2015 to emphasise the period of high backscatter. The backscatter coincides with high chlorophyll-a fluorescence and thus infers an increase in phytoplankton biomass, rather than photo-acclimation.

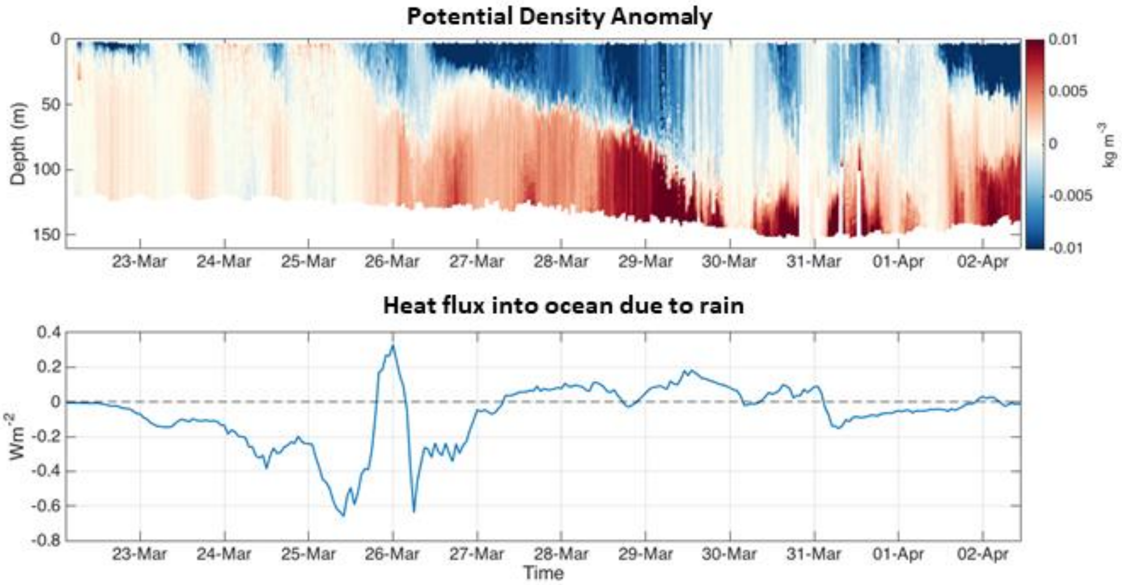

Figure S4: Sensible heat flux into the ocean due to rain. Potential density anomaly (kg m^{-3}; top) and the sensible heat flux due to rain (Wm^{-2}; bottom), whereby positive numbers denote heat being fluxed into the ocean, and was calculated using the Matlab Air-Sea Toolbox and based on the Fortran code by Fairall et al (1996).

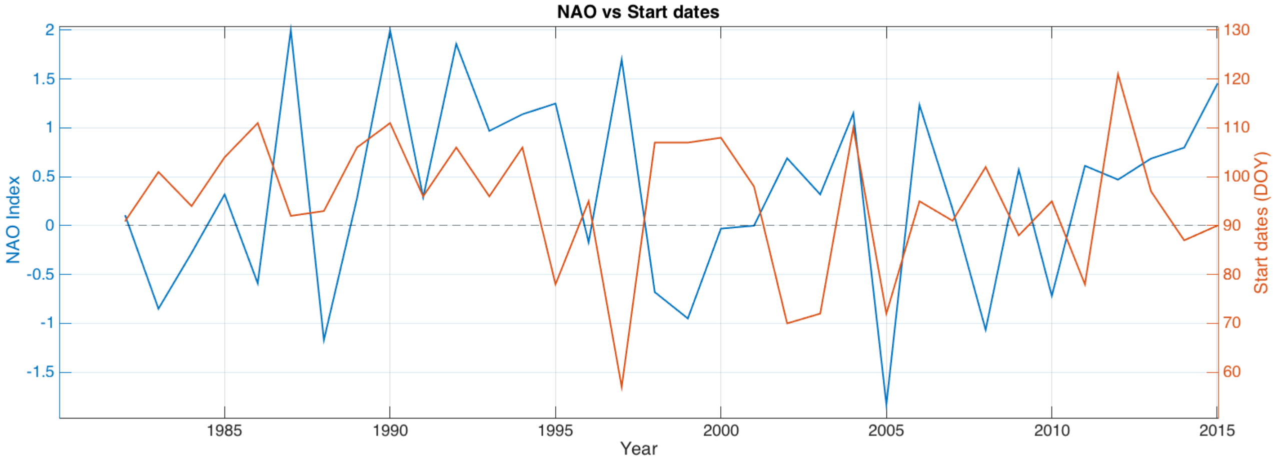

Figure S5: North Atlantic Oscillation (NAO) and stratification onset variability. Comparison between the NAO (blue), averaged across the month of stratification onset to account for the high variability, compared to the onset date of seasonal stratification (red). While there is some evidence of negative correlation from 1996 to 2002, this is not consistent throughout the entire 1982-2015 time period. The North Atlantic Oscillation Index is the 20th Century Reanalysis Version 3 (20CRV3) by Compo et al (2011) which can be accessed here: https://psl.noaa.gov/data/20thC_Rean/timeseries/monthly/NAO/ . (DOI: 10.1002/qj.776)

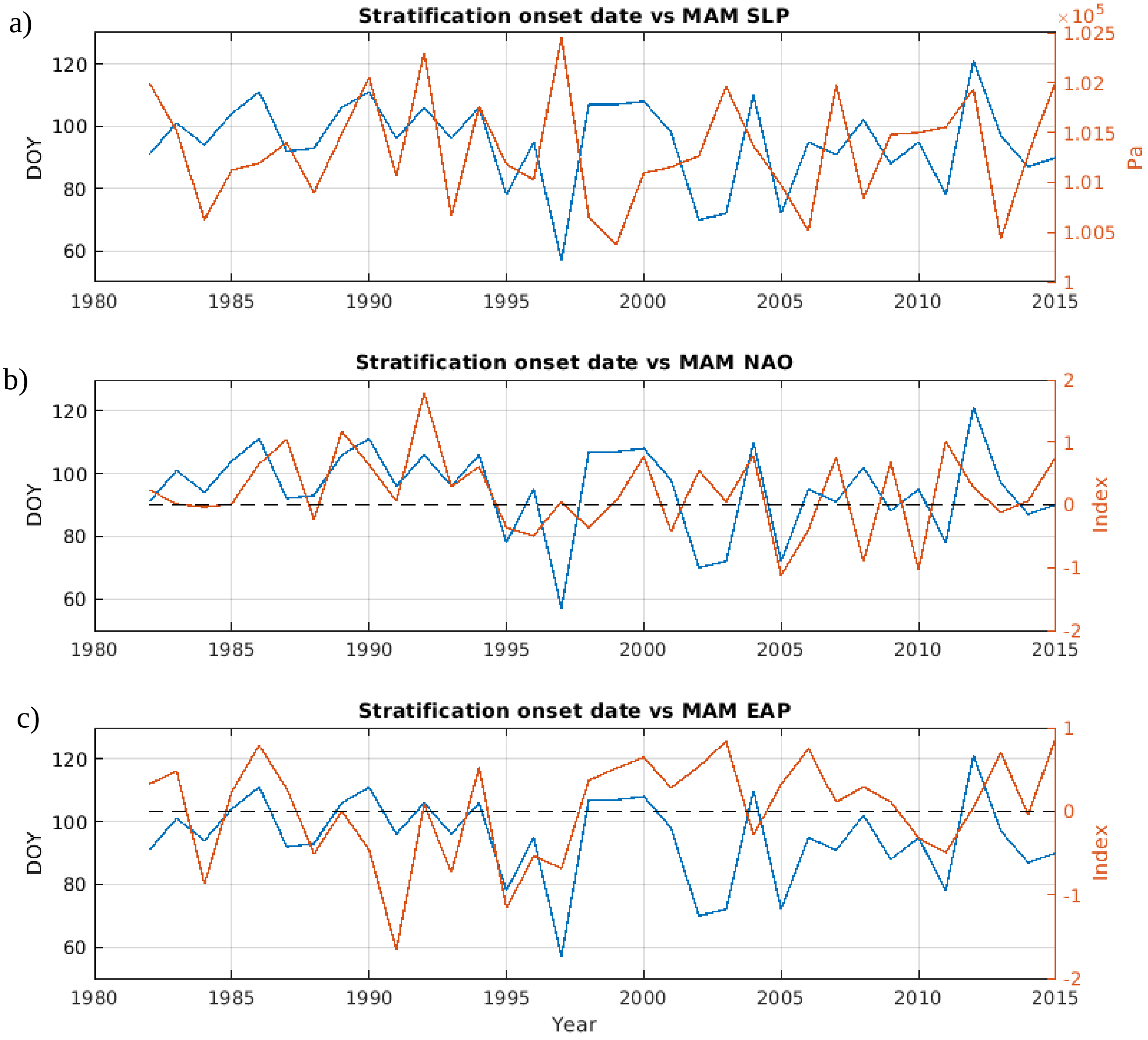

Figure S6: Stratification onset vs climate oscillations. Comparisons of the day of year of stratification onset (blue lines) with a) the March-April-May sea level pressure (MAM SLP), averaged over the Celtic Sea area; b) the March-April-May North Atlantic Oscillation (MAM NAO); and c) the March-April-May East Atlantic Pattern (MAM EAP). No robust relationship can be seen between the stratification onset and the parameter/climate index, however there is some evidence to suggest a shift in the NAO and EAP indices from positive to negative (NAO) and negative to positive (EAP) during the late 1990s, which coincides with the step change in stratification onset date variability, and the phase-shift in the Atlantic Multidecadal Variability (AMV).

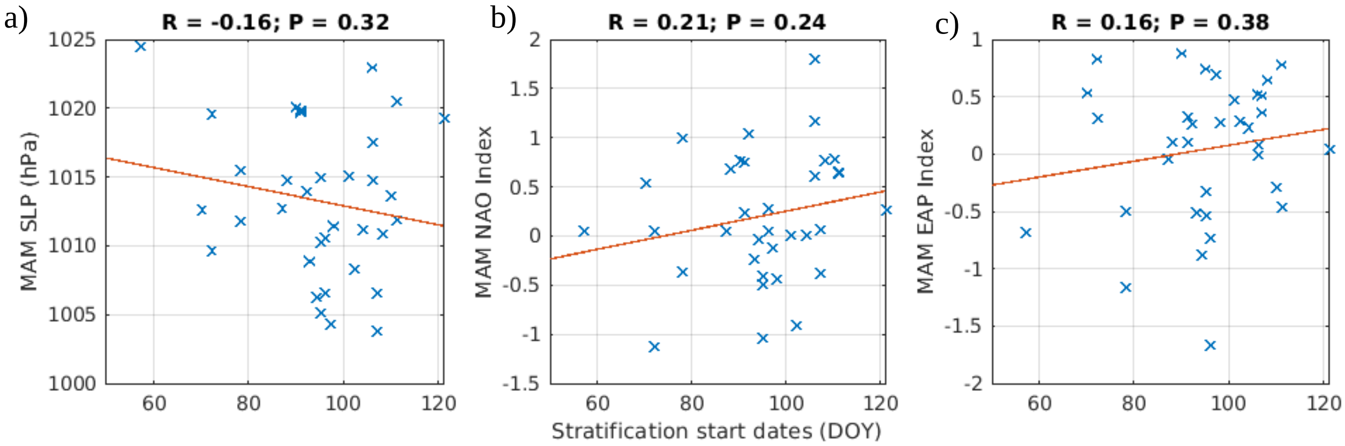

Figure S7: Correlations between stratification onset and climate oscillations. Comparisons between, a) the March-April-May (MAM) SLP, b) the MAM NAO, and c) the MAM EAP and the stratification onset dates (DOY) for the 34-years (blue crosses). The linear relationship is also plotted (red line) as well as the correlation coefficient (R) and the 95% probability (P).

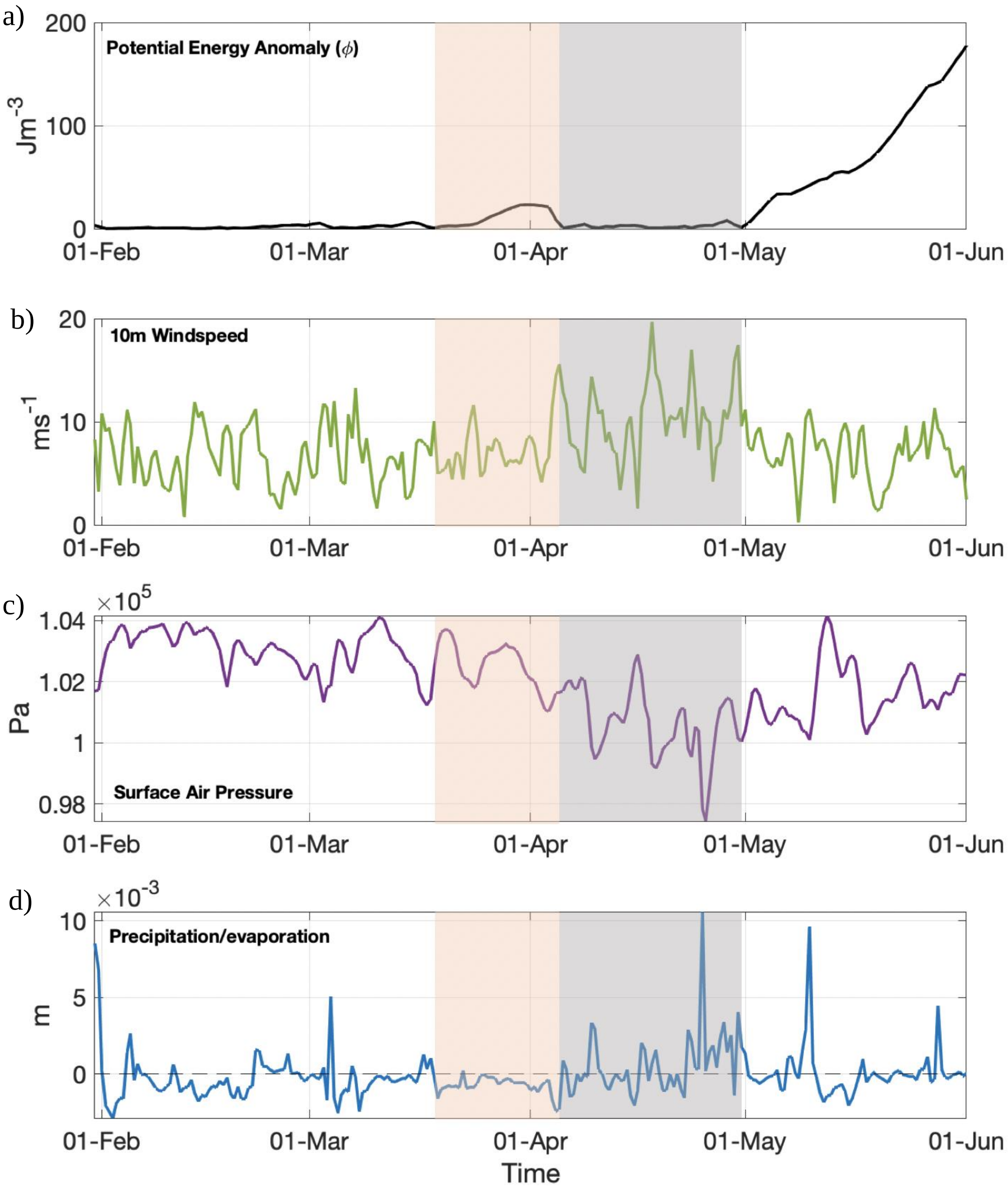

Figure S8: Stratification and meteorological variability in 2012. a) Potential Energy Anomaly (φ; Jm^{-3}), b) 10m wind speed (ms^{-1}), c) surface air pressure (Pa) and d) the amount of water gained (m) from 12hr cumulative precipitation plus evaporation between the 1st February and the 1st June 2012. The orange box indicates the ephemeral stratification event from the 19th March to the 2nd April 2012 that was later homogenised by a series of storms, indicated by the grey box. Seasonal stratification ultimately formed on the 30th April 2012. The potential energy anomaly was calculated from NEMO model data, while meteorological data was sourced from ERA-Interim (Dee et al, 2011).

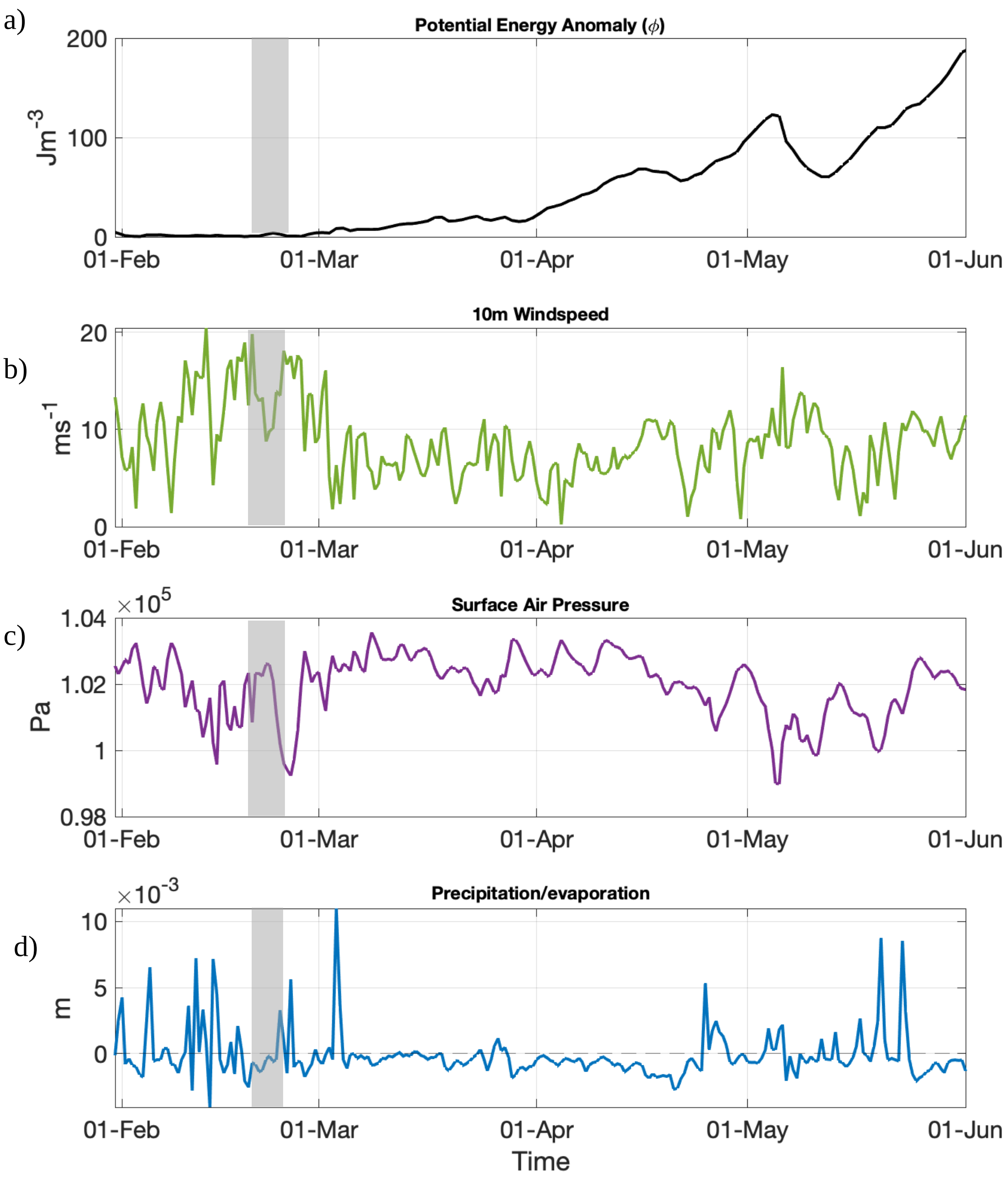

Figure S9: Stratification and meteorological variability in 1997. a) Potential Energy Anomaly (φ; Jm^{-3}), b) 10m wind speed (ms^{-1}), c) surface air pressure (Pa) and d) the amount of water gained (m) from 12hr cumulative precipitation plus evaporation between the 1st February and the 1st June 1997. Seasonal stratification was initiated on the 26th February 1997, at the end of a low-pressure event that occurred on the 21st February for five days (as indicated by the grey box). This was followed by a long period of relatively calm conditions that lasted almost two months. The potential energy anomaly was calculated from NEMO model data, while meteorological data was sourced from ERA-Interim (Dee et al, 2011).

Supplementary Text

Comparisons between the model and glider data confirms the model has a 3-hour offset in stratification timing compared to observations, however this is considered acceptable given the model resolution (7km) and the potential discrepancies between the observed and modelled precipitation over the Celtic Sea. Temperature anomalies were represented well in the model, with consistent maximum and minimum values, yet some of the more subtle switches in surface temperature anomalies between the 27th and 29th March 2015 are not captured. For salinity, the model captures the initial surface decrease in salinity down a depth of 65m (also recorded by the glider), however the modelled salinity is approximately an order of magnitude lower than the glider (likely a result of precipitation forcing data in the model compared to what was observed). Nevertheless, alternating positive and negative salinity anomalies are still well represented over the time period, and therefore we are confident the model represents the small-scale variations from episodic meteorological events.

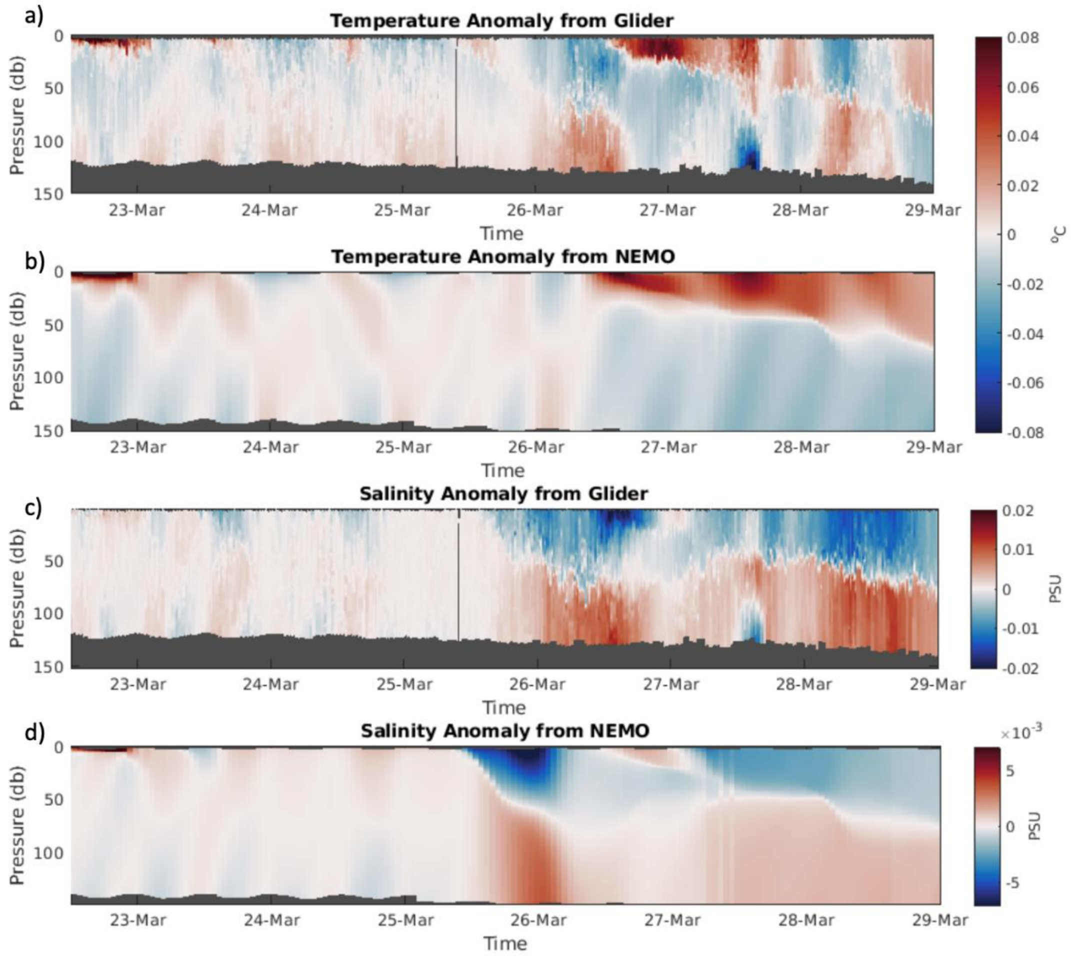

Figure S10: Model validation. Comparisons of glider a) temperature (°C); and c) salinity (PSU) anomalies from the glider against the b) temperature (°C) and d) salinity (PSU) anomalies from the AMM7 model configuration. Anomalies are in relation to the depth-mean average of each profile. The model transect is at the closest time and location to the glider at each time step, within the 7km model bounds. Figure reproduced from Jardine et al, 2022 (accessed here: https://doi.org/10.1029/2021JC017209).

Supplementary References:

Compo, G.P., J.S. Whitaker, P.D. Sardeshmukh, N. Matsui, R.J. Allan, X. Yin, B.E. Gleason, R.S. Vose, G. Rutledge, P. Bessemoulin, S. Brönnimann, M. Brunet, R.I. Crouthamel, A.N. Grant, P.Y. Groisman, P.D. Jones, M. Kruk, A.C. Kruger, G.J. Marshall, M. Maugeri, H.Y. Mok, Ø. Nordli, T.F. Ross, R.M. Trigo, X.L. Wang, S.D. Woodruff, and S.J. Worley, 2011: The Twentieth Century Reanalysis Project. Quarterly J. Roy. Meteorol. Soc., 137, 1-28. DOI: 10.1002/qj.776

Dee, D.P., Uppala, S.M., Simmons, A.J., Berrisford, P., Poli, P., Kobayashi, S., Andrae, U., Balmaseda, M.A., Balsamo, G., Bauer, D.P. and Bechtold, P., 2011. The ERA-Interim reanalysis: Configuration and performance of the data assimilation system. Quarterly Journal of the royal meteorological society, 137(656), pp.553-597.

Fairall, C.W., Bradley, E.F., Rogers, D.P., Edson, J.B. and Young, G.S., 1996. Bulk parameterization of airsea fluxes for tropical ocean-global atmosphere coupled-ocean atmosphere response experiment. Journal of Geophysical Research: Oceans, 101(C2), pp.3747-3764.

Huffman, G.J., E.F. Stocker, D.T. Bolvin, E.J. Nelkin, Jackson Tan (2019), GPM IMERG Final Precipitation L3 Half Hourly 0.1 degree x 0.1 degree V06, Greenbelt, MD, Goddard Earth Sciences Data and Information Services Center (GES DISC), Accessed: [Data Access Date], 10.5067/GPM/IMERG/3B-HH/06

Jardine, J. E., Palmer, M., Mahaffey, C., Holt, J., Wakelin, S., & Artioli, Y. (2022). Climatic controls on the spring phytoplankton growing season in a temperate shelf sea. Journal of Geophysical Research: Oceans, 127, e2021JC017209. https://doi.org/10.1029/2021JC017209

Madec, G., 2015. NEMO ocean engine. Retrieved from https://epic.awi.de/id/eprint/39698/1/NEMO_book_v6039.pdf
